# Pretreatment with Cholecalciferol Alleviates Renal Cellular Stress Response during Ischemia/Reperfusion-Induced Acute Kidney Injury

**DOI:** 10.1155/2019/1897316

**Published:** 2019-03-25

**Authors:** Jun Li, Shen Xu, Jin-Bo Zhu, Jin Song, Biao Luo, Ya-Ping Song, Zhi-Hui Zhang, Yuan-Hua Chen, Zhi-Qiang Zhang, Dong-Dong Xie, De-Xin Yu, De-Xiang Xu

**Affiliations:** ^1^The Second Affiliated Hospital, Anhui Medical University, Hefei, China; ^2^Department of Toxicology, Anhui Medical University, Hefei, China; ^3^Department of Histology and Embryology, Anhui Medical University, Hefei, China; ^4^Laboratory of Environmental Toxicology, Hefei, China

## Abstract

**Background:**

Cellular stress is involved in ischemia/reperfusion- (I/R-) induced acute kidney injury (AKI). This study is aimed at investigating the effects of pretreatment with cholecalciferol on renal oxidative stress and endoplasmic reticulum (ER) stress during I/R-induced AKI.

**Methods:**

I/R-induced AKI was established by cross-clamping renal pedicles for 90 minutes and then reperfusion. In the Chol + I/R group, mice were orally administered with three doses of cholecalciferol (25 *μ*g/kg) at 1, 24, and 48 h before ischemia. Renal cellular stress and kidney injury were measured at different time points after reperfusion.

**Results:**

I/R-induced AKI was alleviated in mice pretreated with cholecalciferol. In addition, I/R-induced renal cell apoptosis, as determined by TUNEL, was suppressed by cholecalciferol. Additional experiment showed that I/R-induced upregulation of renal GRP78 and CHOP was inhibited by cholecalciferol. I/R-induced renal IRE1*α* and eIF2*α* phosphorylation was attenuated by cholecalciferol. Moreover, I/R-induced renal GSH depletion, lipid peroxidation, and protein nitration were blocked in mice pretreated with cholecalciferol. I/R-induced upregulation of renal NADPH oxidases, such as *p47phox*, *gp91phox*, and *nox4*, was inhibited by cholecalciferol. I/R-induced upregulation of heme oxygenase- (HO-) 1, *gshpx* and *gshrd*, was attenuated in mice pretreated with cholecalciferol.

**Conclusions:**

Pretreatment with cholecalciferol protects against I/R-induced AKI partially through suppressing renal cellular stress response.

## 1. Introduction

Acute kidney injury (AKI), defined as a rapid renal dysfunction with severe tubular damage, is a global public health concern associated with high morbidity, mortality, and healthcare costs [1–4]. Renal ischemia/reperfusion (I/R), which induces tubular epithelial cell disruption, is one of the most common causes for AKI [5]. I/R-induced AKI is a frequent event in renal transplantation, especially when the kidney comes from a deceased donor [6]. According to a recent report, oxidative stress played important roles during I/R-evoked AKI [7]. On the other hand, several studies found that renal endoplasmic reticulum (ER) stress was involved in the pathogenesis of I/R-evoked AKI [8–10].

Vitamin D is a lipophilic steroid prohormone. In the past, vitamin D was well known for its classical role on calcium and phosphorus metabolism [11]. Recently, numerous studies have demonstrated that vitamin D plays nonclassical roles by its immune regulation and anti-inflammatory activity [12–14]. Vitamin D3 (VitD3) is the major type of vitamin D. Cholecalciferol, a nonactive form of VitD3, is converted to 25(OH)D3 by cytochrome P450 (CYP)2R1 in the liver and is then converted into 1,25(OH)2D3, the active form of VitD3, by CYP27B1 in the kidney [15]. The actions of 1,25(OH)2D3 are mediated by vitamin D receptor (VDR) [16]. Several studies demonstrated that CYP27B1 and VDR, two key components for VitD3 activity, were highly expressed in human and rodent kidneys [17–19]. Two recent reports indicated that 1,25(OH)2D3 could inhibit renal fibrosis through activating renal VDR [20, 21]. In addition, paricalcitol, an active form of VitD3, suppressed renal inflammatory infiltration in a mouse model of obstructive nephropathy [22]. According to a recent report, pretreatment with cholecalciferol inhibited renal inflammation during lipopolysaccharide- (LPS-) induced AKI [23]. Nevertheless, whether cholecalciferol protects against I/R-induced AKI needs to be determined.

The aim of the present study was to investigate the effect of pretreatment with cholecalciferol on renal oxidative and ER stress during I/R-induced AKI. Our results showed that pretreatment with cholecalciferol protected against I/R-induced AKI and renal dysfunction. We found that pretreatment with cholecalciferol inhibited renal oxidative and ER stress in I/R-induced AKI. We provide evidence that pretreatment with cholecalciferol protects against I/R-induced AKI partially through suppressing renal cellular stress response.

## 2. Materials and Methods

### 2.1. Chemicals and Reagents

Cholecalciferol was from Sigma Chemicals (St. Louis, Missouri). 3-Nitrotyrosine (3-NT), VDR, *β*-actin, HO-1, and HO-2 antibodies and goat anti-rabbit, goat anti-mouse, and anti-goat immunoglobulin secondary antibodies were from Santa Cruz Biotechnology (Santa Cruz, CA). Phospho-eIF2*α* (p-eIF2*α*), phospho-IRE1*α*, NOX4, CHOP, and GRP78 antibodies were from Cell Signaling Technology (Beverley, MA). Chemiluminescent (ECL) detection kits were from Pierce Biotechnology (Rockford, IL). TRI reagent was from the American Molecular Research Center. RNase-free DNase and AMV were from Promega Corporation (Madison, WI). All other reagents were from Sigma Chemical Company (St. Louis, Missouri).

### 2.2. Animals and Treatments

All experimental ICR male mice (12 weeks old, 30-34 g) were from Beijing Vital River whose foundation colonies were all introduced from Charles River Laboratories. All animals were placed in the controlled environment with a temperature of 20-25°C and humidity of 50 ± 5% on a 12 h light-dark cycle and given sufficient food and water all the time. After the end of the experiment, all animals were sacrificed by cervical dislocation. All animal experiments followed the guidelines for humane treatment set by the Association of Laboratory Animal Sciences and the Center for Laboratory Animal Sciences at Anhui Medical University.

### 2.3. Cholecalciferol Pretreatment and Surgical Procedure

A total of 48 mice were randomly divided into 4 groups. General anesthesia was conducted by inhalation of isoflurane (3% vol/vol) mixed with air (1 L/min) in an isoflurane vaporizer (RWD Life Science Ltd., Nanshan Dist., Shenzhen, China). In the I/R and Chol + I/R groups, unilateral ischemia was insured by cross-clamping renal pedicles for 90 minutes in anesthetized mice with contralateral kidneys excisional before the end of the ischemic surgery and the environmental temperature was kept at 30°C-32°C. In the Control and Chol groups, mice were subjected to the same procedure but the renal pedicles were not clamped. In the Chol + I/R and Chol groups, mice were orally administered with cholecalciferol at a dose of 25 *μ*g/kg at 48 hours, 24 hours, and 1 hour before I/R surgery; in the Control and I/R groups, mice were orally administered with the same volume of saline (NS) in the same periods. Six animals in each group were sacrificed 4 hours after reperfusion. Kidneys were collected. Partial renal tissue was fixed in 4% paraformaldehyde for morphological examination and immunohistochemistry (IHC). The remaining renal tissue was used for Western blotting, real-time RT-PCR, and biochemistry. Six animals in each group were sacrificed 24 hours after reperfusion. Blood sera were collected for measurement of renal function. Kidneys were collected for morphological examination.

### 2.4. Biochemistry and Morphological Examination

The renal MDA level was detected by TBARS method according to others [24]. Renal GSH content was determined by Beutler modification method [25]. Renal tissue was fixed in 4% paraformaldehyde to prepare paraffin-embedded tissue sections. The pathological damage was observed by hematoxylin and eosin (H&E). Tubular injury was evaluated in a double-blinded fashion by two different pathologists based on a semiquantitative scale as previously described [26]. Briefly, each cortical tubule showing epithelial cell necrosis and brush border loss was assigned a score of zero for normal, one for loss of brush border or cell necrosis in <25% of tubular cells, two for cell necrosis in 25%-50% of tubular cells, three for cell necrosis in 50%-75% of tubular cells, and four for cell necrosis in >75% of tubular cells. Blood urea nitrogen (BUN) was detected with colorimetric detection kits. Serum creatinine was detected with HPLC.

### 2.5. Real-Time RT-PCR

Real-time RT-PCR was performed as previously described [27]. Briefly, total RNA in kidney tissue was extracted using the TRI reagent. Total RNA (1.0 *μ*g) was treated with RNase-free DNase and reverse transcribed with AMV (Promega). The primers used in the RT-PCR experiments were designed by PubMed's online Primer3 software and synthesized by Life Technologies. The relevant primer sequences are shown in Table 1. The PCR amplification reaction was cyclically amplified with 50 cycles in a three-step process of denaturation, annealing, and extension. The relative ratio of the target gene was calculated using LightCycler 480 software (Roche version 1.5.0).

### 2.6. Immunoblots

Immunoblots were performed as previously described [27]. Briefly, 50 mg renal tissue was used to fabricate renal lysate in 300 *μ*L lysis buffer (50 mM Tris-HCl, pH 7.4, 150 mM NaCl, 1 mM EDTA, 1% Triton X-100, 1% sodium deoxycholate, 0.1% sodium dodecyl sulphate, and 1 mM phenylmethylsulfonyl fluoride) supplemented with a cocktail of protease inhibitors (Roche). For extraction of nuclear protein, kidney homogenate was passed through a 300-mesh sieve and centrifuged at 1000 rpm for 5 minutes. The supernatant was removed and 1 mL PBS was added. The cell pellet was resuspended and centrifuged at 1000 rpm for 5 minutes. The supernatant was then removed. About 300-500 *μ*L of 0.1% NP-40 precooled PBS with a protease inhibitor was added and centrifuged at 10000 rpm for 5 minutes. After the nucleus was resuspended, 1 mL precooled PBS containing 0.1% NP-40 was added. The mixture was homogenized and centrifuged at 10000 rpm for 20 seconds to remove the supernatant. About 100-200 *μ*L RIPA lysates containing protease inhibitors was lysed on ice for 30 minutes. The supernatants were collected and assayed using bicinchoninic acid (BCA) protein assay reagent (Pierce, Rockford, IL, USA) according to the manufacturer's instructions. Using SDS-PAGE electrophoretic separation, the same amount of protein (40-80 *μ*g) was transferred to a polyvinylidene difluoride membrane. The membrane was incubated at room temperature for 2 hours using the following antibodies: HO-1, HO-2, p-eIF2*α*, CHOP, p-IRE1*α*, NOX4, and GRP78. The membranes were washed four times for 10 minutes each time in DPBS containing 0.05% Tween-20 and incubated with goat anti-rabbit IgG or goat anti-mouse antibody for 2 hours. The membranes were then washed four times for 10 minutes each in DPBS containing 0.05% Tween-20, followed by signal development using an ECL detection kit.

### 2.7. Immunohistochemistry (IHC)

For IHC, paraffin-embedded renal sections were deparaffinized and then rehydrated in a graded ethanol series as previously described [28]. After antigen retrieval and quenching of endogenous peroxidase, sections were incubated with GRP78 monoclonal antibody (1 : 200 dilution), VDR monoclonal antibody (1 : 200 dilution), or 3-NT monoclonal antibody (1 : 200 dilution) at 25°C overnight. The color reaction was developed with the HRP-linked polymer detection system (Golden Bridge International, WA, USA) and counterstaining with hematoxylin. 3-NT- and GRP78-positive cells and VDR-positive nuclei were quantified in six random 400x fields of each section from six different mice.

### 2.8. Terminal dUTP Nick-End Labeling (TUNEL) Staining

Apoptosis was detected using an in situ apoptosis detection kit (Promega, Madison, WI) as previously described [27]. Briefly, kidney sections were stained with the TUNEL technology according to the manufacturer's direction. TUNEL-positive cells were analyzed in six random 400x fields of each section from six different mice.

### 2.9. Statistical Analysis

SPSS 19.0 statistical software was used for data analysis. Normally distributed data was expressed as mean ± SEM. The difference between different groups was determined by using ANOVA and the Student-Newman-Keuls post hoc method. The comparison of the mean of independent samples between two groups was performed using *t*-test. *P* < 0.05 is used to indicate statistical significance.

## 3. Results

### 3.1. Pretreatment with Cholecalciferol Activates Renal VDR Signaling

**Figure 1:**
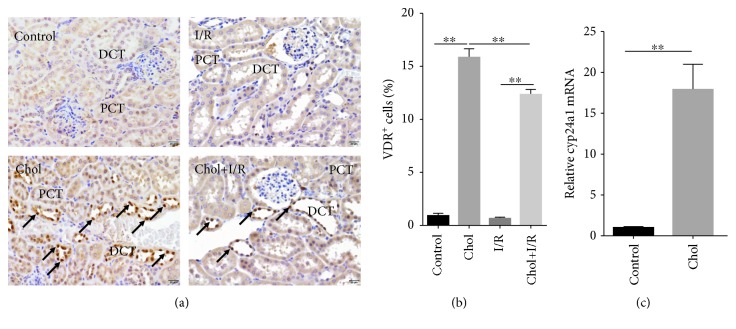
Pretreatment with cholecalciferol activates renal VDR signaling. In the I/R and Chol + I/R groups, mice were conducted with ischemia/reperfusion surgery. In the Chol and Chol + I/R groups, mice were orally administered with three doses of cholecalciferol (25 *μ*g/kg) at 48, 24, and 1 h before I/R surgery. Kidneys were collected with a 4 h period of reperfusion after ischemia surgery. Renal nuclear VDR translocation was analyzed using IHC. Original magnification 400x. (a) VDR^+^ nuclei were distributed among the distal convoluted tubule. (b) The percentage of VDR^+^ nuclei were evaluated among different groups. (c) *Cyp24a1* mRNA was measured using real-time RT-PCR. DCT: distal convoluted tubule; PCT: proximal convoluted tubule. All data were expressed as means ± SEM (*n* = 6). ^∗∗^*P* < 0.01.

The effect of pretreatment with cholecalciferol on renal VDR signaling was detected. As shown in Figure 1(a), VDR^+^ nuclei were mainly distributed in proximal renal tubular cells. As expected, the numbers of renal VDR^+^ nuclei were markedly increased in the Chol group (Figure 1(b)). Of interest, the numbers of renal VDR^+^ nuclei were also elevated in the Chol + I/R group as compared with the I/R group (Figures 1(a) and 1(b)). The effect of pretreatment with cholecalciferol on renal *Cyp24a1* mRNA was then analyzed. As shown in Figure 1(c), *Cyp24a1*, a downstream target gene of VDR, was obviously upregulated in the kidneys of mice pretreated with cholecalciferol.

### 3.2. Pretreatment with Cholecalciferol Alleviates I/R-Induced AKI

**Figure 2:**
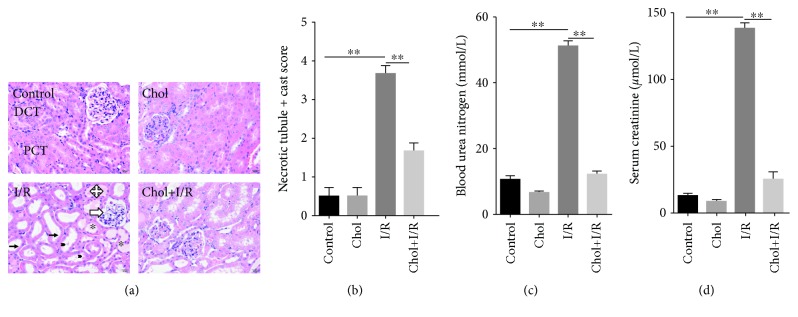
Pretreatment with cholecalciferol alleviates I/R-induced AKI. In the I/R and Chol + I/R groups, mice were conducted with ischemia/reperfusion surgery. In the Chol and Chol + I/R groups, mice were orally administered with three doses of cholecalciferol (25 *μ*g/kg) at 48, 24, and 1 h before I/R surgery. Kidney and blood samples were collected with a 24 h period of reperfusion after ischemia surgery. (a, b) Renal histopathology was evaluated. (a) Long thin black arrows indicate intact brush border; hollow arrows show dilation of renal capsule cavity; cross arrow shows flattened renal tubule epithelial cells; black arrowheads point cell debris of renal tubule epithelial cells; asterisk shows hyaline accumulation. DCT: distal convoluted tubule; PCT: proximal convoluted tubule. (b) Semiquantitative evaluation of tubular injury on a zero to four scale among different groups. (c, d) Renal function was measured. (c) BUN. (d) Serum creatinine. All data were expressed as means ± SEM (*n* = 6). ^∗∗^*P* < 0.01.

The effect of pretreatment with cholecalciferol on I/R-induced AKI is presented in Figure 2. As expected, 24 hours after reperfusion, obvious pathological lesions in the renal cortex and lateral medulla, such as dilation of renal capsule cavity, glomerular cyst expansion, vacuolization of tubular epithelial cells, and focal necrosis, were observed in renal tubules (Figure 2(a)). Interestingly, pretreatment with cholecalciferol attenuated I/R-induced renal pathological injury (Figures 2(a) and 2(b)). The effect of pretreatment with cholecalciferol on I/R-induced renal dysfunction was then evaluated. Twenty-four hours after reperfusion, the level of serum creatinine was significantly elevated (Figure 2(c)). Accordingly, the level of BUN was increased (Figure 2(d)). Interestingly, pretreatment with cholecalciferol almost completely inhibited I/R-induced acute renal dysfunction (Figures 2(c) and 2(d)).

### 3.3. Pretreatment with Cholecalciferol Inhibits Renal Cell Apoptosis in I/R-Induced AKI

**Figure 3:**
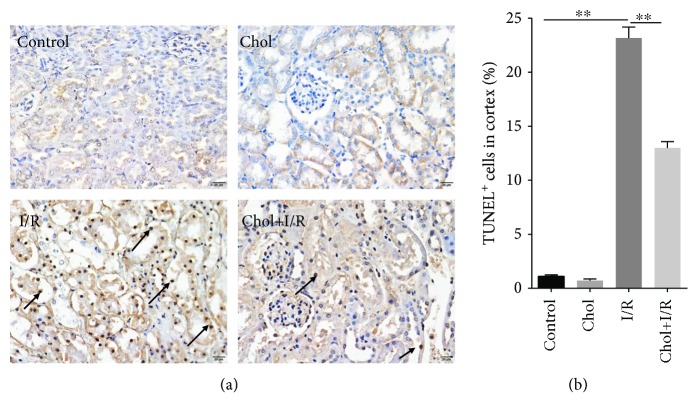
Pretreatment with cholecalciferol inhibits I/R-induced renal cell apoptosis. In the I/R and Chol + I/R groups, mice were conducted with ischemia/reperfusion surgery. In the Chol and Chol + I/R groups, mice were orally administered with three doses of cholecalciferol (25 *μ*g/kg) at 48, 24, and 1 h before I/R surgery. Kidney samples were collected with a 4 h period of reperfusion after ischemia surgery. (a) Renal cell apoptosis was detected by TUNEL assay. Arrows show TUNEL^+^ cells in renal cortical and medullary tubules. Representative photomicrographs were shown from different groups. Original magnification 400x. (b) TUNEL^+^ cells were analyzed. All data were expressed as means ± SEM (*n* = 6). ^∗∗^*P* < 0.01.

The effect of pretreatment with cholecalciferol on I/R-induced renal cell apoptosis was analyzed. As shown in Figure 3(a), many TUNEL-positive cells were observed in the cortex of mouse kidneys 4 hours after reperfusion. Interestingly, pretreatment with cholecalciferol obviously alleviated renal cortical tubular epithelial cell apoptosis during I/R-induced AKI (Figures 3(a) and 3(b)).

### 3.4. Pretreatment with Cholecalciferol Attenuates Renal ER Stress in I/R-Induced AKI

**Figure 4:**
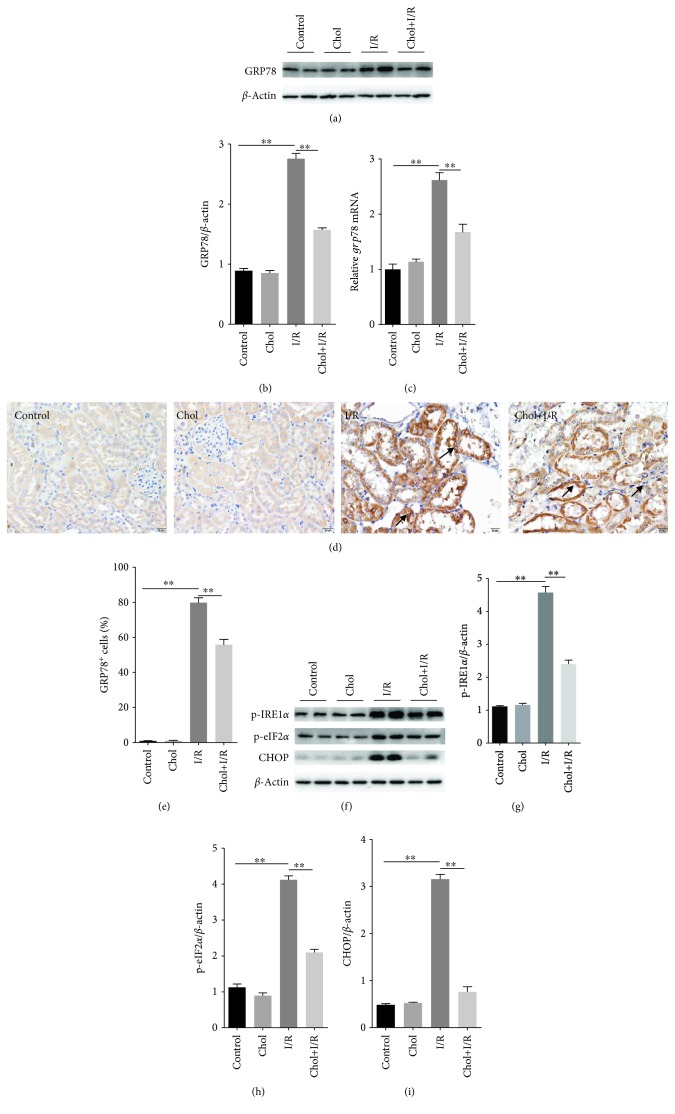
Pretreatment with cholecalciferol alleviates renal ER stress during I/R-induced AKI. In the I/R and Chol + I/R groups, mice were conducted with ischemia/reperfusion surgery. In the Chol and Chol + I/R groups, mice were orally administered with three doses of cholecalciferol (25 *μ*g/kg) at 48, 24, and 1 h before I/R surgery. Kidney samples were collected with a 4 h period of reperfusion after ischemia surgery. (a, b) Renal GRP78 was measured using immunoblots. (a) A representative gel for GRP78 and *β*-actin was shown. (b) GRP78/*β*-actin. (c) Renal *grp78* mRNA was measured using real-time RT-PCR. (d, e) Renal GRP78 was analyzed by immunohistochemistry. (d) Representative photomicrographs of renal specimens from different groups are presented. Original magnification 400x. Renal GRP78 was mainly distributed in cortical tubular epithelial cells. (e) GRP78^+^ cells were analyzed. (f–i) Renal p-IRE1*α*, p-eIF2*α*, and CHOP were measured using immunoblots. (f) A representative gel for p-IRE1*α*, p-eIF2*α*, CHOP, and *β*-actin was shown. (g) p-IRE1*α*/*β*-actin. (h) p-eIF2*α*/*β*-actin. (i) CHOP/*β*-actin. All data were expressed as means ± SEM (*n* = 6). ^∗∗^*P* < 0.01.

The effect of pretreatment with cholecalciferol on I/R-induced upregulation of renal GRP78 was analyzed. As expected, renal GRP78 protein was markedly upregulated at 4 hours after reperfusion (Figures 4(a) and 4(b)). In addition, the renal *grp78* mRNA level was obviously elevated 4 hours after reperfusion (Figure 4(c)). IHC showed that renal GRP78 was mainly distributed in cortical tubular epithelial cells (Figure 4(d)). As shown in Figures 4(a)–4(c), pretreatment with cholecalciferol significantly attenuated I/R-induced upregulation of renal GRP78 protein and mRNA. In addition, pretreatment with cholecalciferol significantly attenuated I/R-induced elevation of GRP78^+^ cells in cortical tubular epithelial cells (Figures 4(d) and 4(e)). Renal IRE1*α* phosphorylation during I/R-induced AKI was measured. As shown in Figures 4(f) and 4(e), renal p-IRE1*α* was obviously elevated 4 hours after reperfusion. Interestingly, pretreatment with cholecalciferol inhibited I/R-induced renal IRE1*α* phosphorylation (Figures 4(f) and 4(e)). Finally, renal eIF2*α* phosphorylation and CHOP expression during I/R-induced AKI were analyzed. As shown in Figures 4(f) and 4(h), renal p-eIF2*α* was significantly elevated 4 hours after reperfusion. Moreover, renal CHOP expression was upregulated 4 hours after reperfusion (Figures 4(f) and 4(i)). Pretreatment with cholecalciferol suppressed renal eIF2*α* phosphorylation and CHOP upregulation during I/R-induced AKI **(**Figures 4(f), 4(h), and 4(i)).

### 3.5. Pretreatment with Cholecalciferol Attenuates Renal Oxidative Stress during I/R-Induced AKI

**Figure 5:**
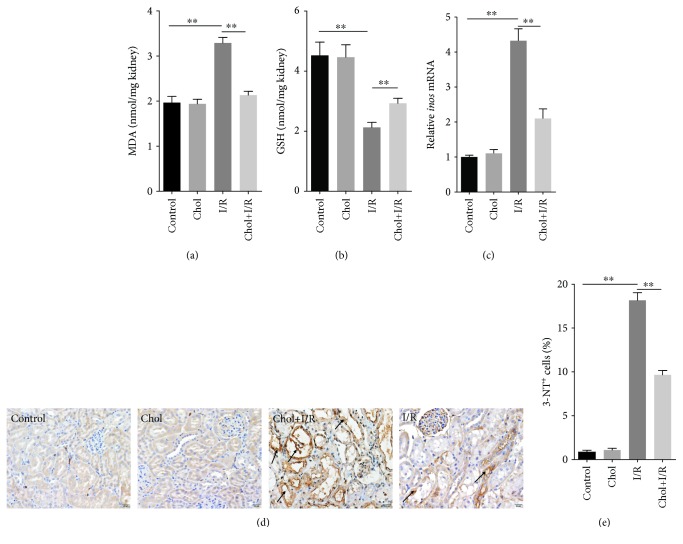
Pretreatment with cholecalciferol alleviates renal GSH depletion, lipid peroxidation, and protein nitration during I/R-induced AKI. In the I/R and Chol + I/R groups, mice were conducted with ischemia/reperfusion surgery. In the Chol and Chol + I/R groups, mice were orally administered with three doses of cholecalciferol (25 *μ*g/kg) at 48, 24, and 1 h before I/R surgery. Kidney samples were collected with a 4 h period of reperfusion after ischemia surgery. (a) Renal MDA content. (b) Renal GSH content. (c) Renal *inos* mRNA was measured using real-time RT-PCR. (d, e) Renal 3-NT residue was measured using immunohistochemistry. (d) Representative photomicrographs of renal specimens from different groups are presented. Original magnification 400x. (e) 3-NT^+^ cells were analyzed. All data were expressed as means ± SEM (*n* = 6). ^∗∗^*P* < 0.01.

The effect of pretreatment with cholecalciferol on I/R-induced renal lipid peroxidation and GSH depletion was investigated. As shown in Figure 5(a), renal MDA, a marker of lipid peroxidation, was markedly elevated 4 hours after reperfusion. By contrary, the renal GSH content was obviously reduced 4 hours after reperfusion (Figure 5(b)). Interestingly, pretreatment with cholecalciferol significantly attenuated I/R-induced renal MDA elevation and GSH depletion (Figures 5(a) and 5(b)). The effect of pretreatment with cholecalciferol on I/R-induced renal *inos* mRNA upregulation is presented in Figure 5(c). As expected, renal *inos* mRNA was obviously upregulated 4 hours after reperfusion. Pretreatment with cholecalciferol significantly attenuated I/R-induced upregulation of renal *inos* mRNA. Finally, the effect of pretreatment with cholecalciferol on I/R-induced renal protein nitration was analyzed by IHC. As shown in Figure 5(d), renal 3-NT residue, a marker of protein nitration, was mainly distributed in tubular epithelial cells of the renal cortex 4 hours after reperfusion. Interestingly, pretreatment with cholecalciferol almost completely blocked renal protein nitration during I/R-induced AKI (Figures 5(d) and 5(e)).

### 3.6. Pretreatment with Cholecalciferol Regulates Expression of Renal Antioxidant Enzymes during I/R-Induced AKI

**Figure 6:**
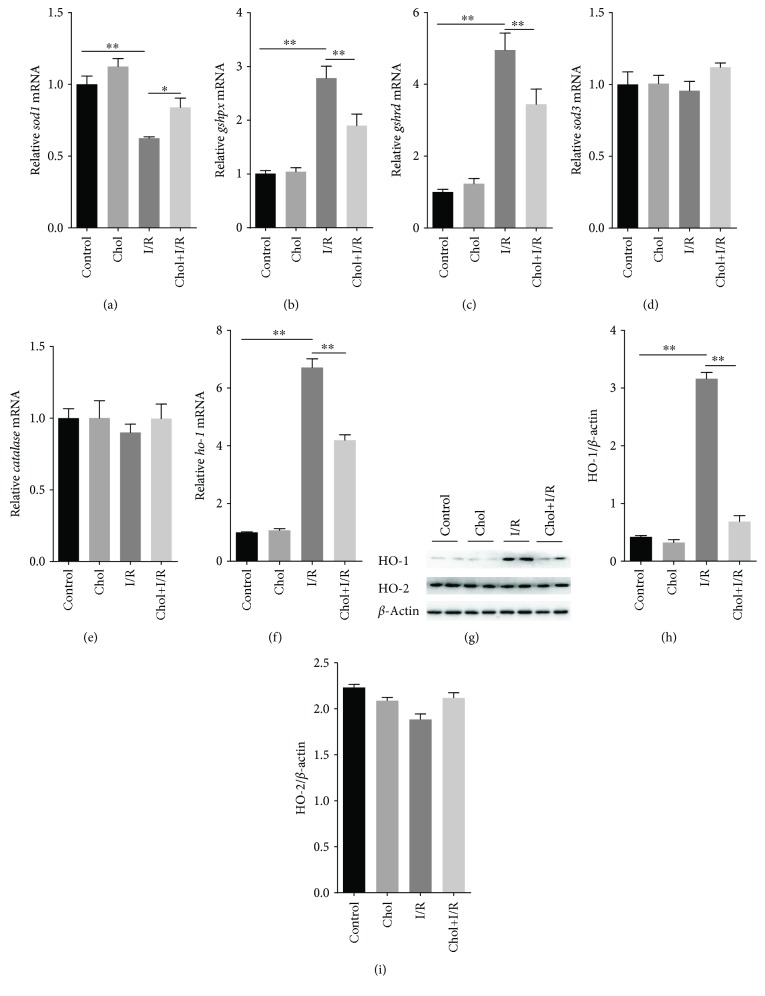
Pretreatment with cholecalciferol regulates the expression of renal antioxidant enzymes during I/R-induced AKI. In the I/R and Chol + I/R groups, mice were conducted with ischemia/reperfusion surgery. In the Chol and Chol + I/R groups, mice were orally administered with three doses of cholecalciferol (25 *μ*g/kg) at 48, 24, and 1 h before I/R surgery. Kidney samples were collected with a 4 h period of reperfusion after ischemia surgery. (a–e) Renal *sod1*, *gshpx*, *gshrd*, *sod3*, and *catalase* mRNAs were measured using real-time RT-PCR. (a) *sod1*; (b) *gshpx*; (c) *gshrd*; (d) *sod3*; (e) *catalase*. (f) Renal *ho-1* mRNA was measured using real-time RT-PCR. (g–i) Renal HO-1 and HO-2 proteins were measured using immunoblots. (g) A representative gel for HO-1, HO-2, and *β*-actin was shown. (h) HO-1/*β*-actin. (i) HO-2/*β*-actin. All data were expressed as means ± SEM (*n* = 6). ^∗^*P* < 0.05; ^∗∗^*P* < 0.01.

The effect of pretreatment with cholecalciferol on the expression of renal antioxidant enzymes is presented in Figures 6(a)–6(e). Although I/R had little effect on the expression of renal *sod3* and *catalase* (Figures 6(d) and 6(e)), renal *sod1* mRNA was downregulated 4 hours after reperfusion (Figure 6(a)). By contrast, renal *gshpx* and *gshrd* mRNAs were upregulated 4 hours after reperfusion (Figures 6(b) and 6(c)). Of interest, pretreatment with cholecalciferol attenuated renal *sod1* downregulation during I/R-induced AKI (Figure 6(a)). In addition, pretreatment with cholecalciferol alleviated renal *gshpx* and *gshrd* upregulation during I/R-induced AKI (Figures 6(b) and 6(c)). The effect of pretreatment with cholecalciferol on I/R-induced renal HO-1 was then analyzed. Although I/R had little effect on renal HO-2 (Figures 6(g) and 6(i)), renal *ho-1* mRNA was obviously upregulated 4 hours after reperfusion (Figure 6(f)). The level of renal HO-1 protein was accordingly elevated 4 hours after reperfusion (Figures 6(g) and 6(h)). Of interest, pretreatment with cholecalciferol obviously attenuated I/R-induced upregulation of renal HO-1 (Figures 6(g) and 6(h)).

### 3.7. Pretreatment with Cholecalciferol Attenuates Upregulation of Renal NADPH Oxidases during I/R-Induced AKI

**Figure 7:**
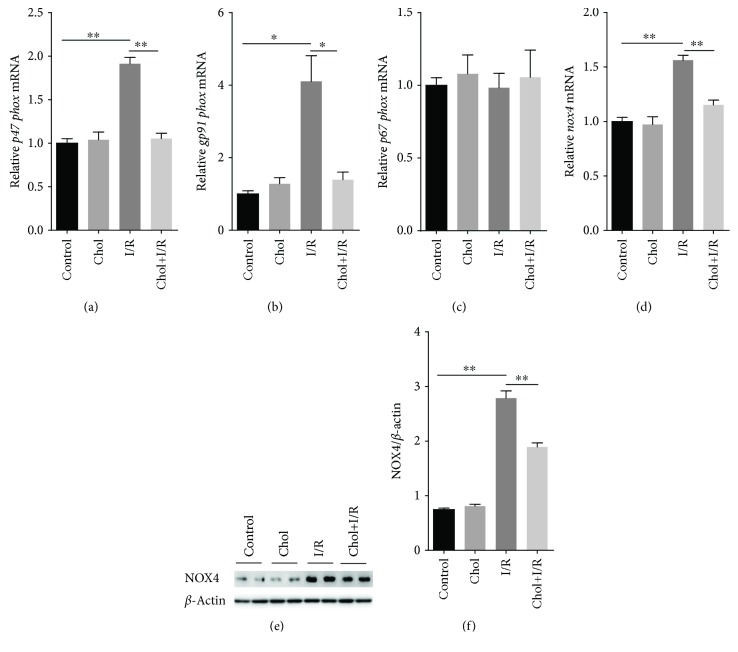
Pretreatment with cholecalciferol inhibits upregulation of renal NADPH oxidases during IR-induced AKI. In the I/R and Chol + I/R groups, mice were conducted with ischemia/reperfusion surgery. In the Chol and Chol + I/R groups, mice were orally administered with three doses of cholecalciferol (25 *μ*g/kg) at 48, 24, and 1 h before I/R surgery. Kidney samples were collected with a 4 h period of reperfusion after ischemia surgery. (a–d) Renal NADPH oxidase subunits were measured using real-time RT-PCR. (a) *p47phox*; (b) *gp91phox*; (c) *p67phox*; (d) *nox4*. (e, f) Renal NOX4 protein was measured using immunoblots. (e) A representative gel for NOX4 and *β*-actin was shown. (f) NOX4/*β*-actin. All data were expressed as means ± SEM (*n* = 6). ^∗^*P* < 0.05; ^∗∗^*P* < 0.01.

The effect of pretreatment with cholecalciferol on the expression of renal NADPH oxidases was analyzed. Although I/R had little effect on *p67phox* mRNA (Figure 7(c)), renal *p47phox* and *gp91phox*, two NADPH oxidase subunits, were upregulated 4 hours after reperfusion (Figures 7(a) and 7(b)). Moreover, renal *nox4* mRNAs were upregulated 4 hours after reperfusion (Figure 7(d)). The level of renal NOX4 protein was accordingly elevated 4 hours after reperfusion (Figures 7(e) and 7(f)). Of interest, pretreatment with cholecalciferol inhibited I/R-induced renal *p47phox* and *gp91phox* (Figures 7(a) and 7(b)). In addition, pretreatment with cholecalciferol attenuated I/R-induced upregulation of renal NOX4 (Figures 7(e) and 7(f)).

## 4. Discussion

Two recent reports found that pretreatment with cholecalciferol protected against sepsis-induced AKI in mice [23, 29]. Additional study showed that pretreatment with cholecalciferol protected against I/R-induced hepatic injury in rats [30]. In the present study, we investigated the effects of pretreatment with cholecalciferol on I/R-induced AKI in mice. Our findings indicated that pretreatment with cholecalciferol alleviated I/R-induced renal pathological injury and acute renal dysfunction. In addition, pretreatment with cholecalciferol inhibited cortical tubular epithelial cell apoptosis during I/R-induced AKI. These results suggest that pretreatment with cholecalciferol protects against I/R-evoked AKI.

Recently, several reports indicate that renal ER stress is involved in the process of I/R-evoked AKI [8–10]. ER stress is mediated by three transmembrane ER-resident stress sensors including inositol-requiring enzyme 1 (IRE1), PKR-like ER kinase (PERK), and activating transcription factor (ATF6) [31]. Under ER stress, GRP78, an ER molecular chaperone, dissociates from all three sensors and initiates unfolded protein response (UPR) [32]. Indeed, the present study showed that renal GRP78 was upregulated during I/R-induced AKI. Moreover, the level of renal p-IRE1*α*, a key molecule of the IRE1 pathway, was markedly increased 4 hours after reperfusion. In addition, the levels of renal p-eIF2*α* and CHOP, two downstream molecules of the PERK pathway, were obviously elevated during I/R-induced AKI. An in vivo study found that VDR activation inhibited ER stress in I/R-induced myocardial injury [33]. An in vitro report showed that active VitD3 suppressed ER stress in tunicamycin-treated human umbilical endothelial cells [34]. The present study found that pretreatment with cholecalciferol attenuated I/R-induced upregulation of renal GRP78. Moreover, pretreatment with cholecalciferol inhibited I/R-induced phosphorylation of renal IRE1*α* and eIF2*α*. In addition, pretreatment with cholecalciferol alleviated I/R-induced upregulation of CHOP in the kidney. These results suggest that pretreatment with cholecalciferol protects against I/R-evoked AKI partially through inhibiting renal ER stress.

Accumulating evidence suggests that oxidative stress plays important roles in I/R-induced AKI [35]. Antioxidants could effectively protect against I/R-induced AKI [7, 36–38]. Several in vivo and in vitro experiments found that active VitD3 had antioxidant activities [39–41]. A recent report showed that pretreatment with cholecalciferol attenuated renal oxidative stress during LPS-induced AKI [29]. In the present study, we further analyzed the effects of pretreatment with cholecalciferol on renal oxidative stress during I/R-induced AKI. As expected, the renal GSH content was obviously decreased in I/R-treated mice. By contrast, renal MDA, a marker of lipid peroxidation, was markedly increased in I/R-treated mice. In addition, renal *inos* mRNA expression was upregulated 4 hours after reperfusion. Accordingly, 3-NT residue, a marker of protein nitration, was observed in tubular epithelial cells of the renal cortex in the I/R group. Interestingly, pretreatment with cholecalciferol attenuated I/R-induced renal GSH depletion and lipid peroxidation. Moreover, pretreatment with cholecalciferol inhibited I/R-induced *inos* mRNA upregulation and protein nitration in the kidneys. These results provide evidences that pretreatment with cholecalciferol protects against I/R-evoked AKI, at least partially, through inhibiting renal oxidative stress.

NADPH oxidases, which are composed of several membrane-associated subunits including p22phox, p47phox, gp91phox, and p67phox, are important enzymatic sources of cellular ROS in the pathogenesis I/R-evoked AKI [42, 43]. Several studies indicate that antioxidant enzymes, such as HO-1 and SOD, protect against I/R-evoked AKI [44–49]. A recent study showed that pretreatment with active VitD3 suppressed the NADPH oxidase NOX2 (also termed gp91phox) subunit during I/R-induced brain injury [50]. The present study found that the expression of renal *gp91phox*, *p47phox*, and *nox4*, three NADPH oxidase subunits, was upregulated in I/R-induced AKI. In addition, the level of renal NOX4 protein was elevated 4 hours after reperfusion. On the other hand, the levels of renal HO-1 and GSH metabolic enzymes were elevated during I/R-induced AKI. By contrast, renal *sod1* mRNA was downregulated 4 hours after reperfusion. In the present study, we further analyzed the effects of pretreatment with cholecalciferol on the expression of NADPH oxidases and antioxidant enzymes in the kidneys. Our results showed that pretreatment with cholecalciferol suppressed I/R-induced upregulation of NAPDH oxidases in the kidneys. Although cholecalciferol alone had little effect on renal antioxidant enzymes, it attenuated I/R-induced upregulation of renal HO-1 and GSH metabolic enzymes. In addition, pretreatment with cholecalciferol reversed I/R-induced downregulation of renal *sod1* mRNA. These results suggest that cholecalciferol suppresses I/R-induced renal oxidative stress, possibly through inhibiting renal NAPDH oxidases and probably through regulating renal antioxidant enzyme genes.

Several epidemiological reports showed that the low vitamin D status was associated with AKI in critically ill patients [51, 52]. According to a recent report, the low vitamin D status promoted the progression of chronic kidney disease after I/R-evoked AKI [53]. Indeed, an earlier study showed that pretreatment with paricalcitol, an active form of VitD3, inhibited I/R-induced renal inflammation through upregulating cyclooxygenase-2 and prostaglandin E2 [54]. A recent study indicated that pretreatment with paricalcitol attenuated I/R-induced AKI through the prostaglandin E2 receptor EP4 pathway [55]. In the present study, we found that pretreatment with cholecalciferol prevented mice from I/R-induced AKI partially through suppressing renal cellular stress. Therefore, VitD3 may be used as a potential protective agent for clinical prevention and therapy especially in renal transplantation patients.

In summary, the present study investigated the effects of pretreatment with cholecalciferol on I/R-induced AKI in mice. Our results showed that pretreatment with cholecalciferol protected against I/R-evoked AKI. We found that pretreatment with cholecalciferol inhibited renal oxidative and ER stress through regulating renal NAPDH oxidase and antioxidant enzyme genes. We provide additional evidences that pretreatment with cholecalciferol protects against I/R-induced AKI partially through suppressing renal cellular stress response.

## Figures and Tables

**Table 1:** Primers for real-time RT-PCR.

Genes	Sequence	Length
*18S*	Forward: 5′-GTAACCCGTTGAACCCCATT-3′	151
	Reverse: 5′-CCATCCAATCGGTAGTAGCG-3′	

*sod1*	Forward: 5′-GCGATGAAAGCGGTGTGCGTG-3′	143
	Reverse: 5′-TGGACGTGGAACCCATGCTGG-3′	

*sod3*	Forward: 5′-CTGGCCGAGACAACACTGACCT-3′	68
	Reverse: 5′-GCGACGACGGTTCTGGTCTCAC-3	

*gshpx*	Forward: 5′-GGTGGTGCTCGGTTTCCCGT-3′	113
	Reverse: 5′-AATTGGGCTCGAACCCGCCAC-3′	

*gshrd*	Forward: 5′-GGGATGCCTATGTGAGCCGC-3′	120
	Reverse: 5′-TGACTTCCACCGTGGGCCGA-3′	

*p47phox*	Forward: 5′-CCAGGGCACTCTCACTGAATA-3′	100
	Reverse: 5′-ATCAGGCCGCACTTTGAAGAA-3′	

*gp91phox*	Forward: 5′-GGGAACTGGGCTGTGAATGA-3′	147
	Reverse: 5′-CAGTGCTGACCCAAGGAGTT-3′	

*p67phox*	Forward: 50-GCTGCGTGAACACTATCCTGG-3′	136
	Reverse: 50-AGGTCGTACTTCTCCATTCTGTA-3′	

*nox4*	Forward: 50-CCAAATGTTGGGCGATTGTGT-3′	133
	Reverse: 50-TCCTGCTAGGGACCTTCTGT-3′	

*inos*	Forward: 50-GCTCGCTTTGCCACGGACGA-3′	146
	Reverse: 50-AAGGCAGCGGGCACATGCAA-3′	

*Catalase*	Forward: 50-CGCGCTCGAGTGGCCAACT-3′	107
	Reverse: 50-TGCTGCTCTGGTGCGCTGAA-3′	

*ho-1*	Forward: 5′-CGTCACTTCGTCAGAGGCCTGC-3′	75
	Reverse: 5′-TCTGGGGTTTCCCTCGGGGTG-3′	

*grp78*	Forward: 5′-CTGGCCGAGACAACACTGACCT-3′	68
	Reverse: 5′-GCGACGACGGTTCTGGTCTCAC-3′	

## Data Availability

The data used to support the findings of this study are available from the corresponding author upon request.

## Abbreviations

DCT: Distal convoluted tubule
GSH: Glutathione
IL: Interleukin
I/R: Ischemia/reperfusion
MDA: Malondialdehyde
PCT: Proximal convoluted tubule
ROS: Reactive oxygen species
TUNEL: Terminal dUTP nick end labeling
VDR: Vitamin D receptor.
